# Analytics and Applications of Audio and Image Sensing Techniques

**DOI:** 10.3390/s22218443

**Published:** 2022-11-03

**Authors:** Alicja Wieczorkowska

**Affiliations:** Department of Multimedia, Polish-Japanese Academy of Information Technology, 02-008 Warsaw, Poland; alicja@poljap.edu.pl

## 1. Introduction

Nowadays, with numerous sensors placed everywhere around us, we can obtain signals collected from a variety of environment-based sensors, including the ones placed on the ground, cased in the air or water, etc. These sensors can produce various signals, such as audio, image, video, infrared data, and so on. The obtained signals are usually processed using dedicated software, utilizing the output of these sensors. The construction of optimized sensors, the development of the theory that can he applied for signal representation and analysis, as well as the design of software for the processing of the obtained data represent broadly explored research topics. Therefore, recent advances in these areas are of interest for many researchers, and for the users of sensing techniques.

The Special Issue entitled “Analytics and Applications of Audio and Image Sensing Techniques” is focused on original research involving the use of various audio and image sensing devices, both simultaneously and separately. It collected a diverse set of papers that span a wide range of analyses and possible applications.

Topics for the Special Issue included the following:

- Digital signal processing;
- Audio signal analysis;
- Image analysis;
- Pattern recognition.

Altogether, 17 papers have been included in this Special Issue.

## 2. Overview of the Contributions

The papers presented in this Special Issue cover a broad range of topics, from the theory of signal analysis, to practical applications of audio and image sensing techniques. Audio signals discussed in these papers cover both speech and music, including measurements of airflow in organ pipes. Image and video data analyzed in the presented papers include microscope images and infrared images, as well as face images. All papers are briefly presented below.

The paper [1] by A. Stepanov proposes a modified wavelet synthesis algorithm for the continuous wavelet transform (CWT). The author proposes to use splines and artificial neural networks for continuous wavelet synthesis. This algorithm provides a guaranteed accuracy in approximating variable signal samples with the mother wavelet. The invertibility of the CWT while using synthesized wavelets is also demonstrated in the paper.

### 2.1. Contributions in the Field of Audio Signal

Six contributions in this Special Issue are dedicated to audio signal, including both music and speech.

The paper [2] by M. Blaszke and B. Kostek addresses the problem of musical instrument identification in audio recordings. Mixes of sounds of four instrument were investigated, namely bass, drums, guitar, and piano. The model consisting of a set of convolutional neural networks (CNN) was applied, with an individual CNN used for each instrument. The efficiency of this model ranged from 0.86 for the guitar to 0.99 for drums, with various metrics used for evaluating the results. The proposed model can be easily extended to identify more instruments, by adding more networks in the model.

P. Wrzeciono in his paper [3] presents his work on finding the original tuning (temperament) and the reference pitch which allowed the reconstruction of pipes in the chest organ from the 17th century, found in Kamień Pomorski (West Pomerania, Poland). This process had to rely basically on the original picture of organ pipes, found on the instrument’s doors, and historical sources that allowed estimating the temperature in this region in 17th century, as the temperature determines the speed of sound in air and the frequency of the sound wave generated by a pipe.

The sound of organ pipes is also addressed in the paper [4] by D. Węgrzyn, P. Wrzeciono, and A. Wieczorkowska. An obstacle placed near a flue pipe’s mouth influences the pitch of the generated sound, and this may happen when the pipe is very close to the organ case, or other pipes. Interval calculus was applied to obtain non-invasive measurements. Turbulent air flow is needed to generate sound in organ pipes, thus the authors analyze the Reynolds number, determining whether the fluid motion is turbulent or not, and the Strouhal number, depending on the pitch of the sound generated by turbulent flow.

P. Odya, J. Kotus, A. Kurowski, and B. Kostek in [5] analyzed rate of speech in reverberant conditions. As a result, the authors indicated the maximum speech rate required to achieve sentence intelligibility for various acoustic spaces, for Polish language. Acoustics tests were carried out, with participants with normal hearing. Slowing down the speech or speech signal, as proposed in this paper, may improve intelligibility in spaces with high reverberation.

In the next paper [6], A. Kurowski, J. Kotus, P. Odya, and B. Kostek propose a novel method for assessing the intelligibility of nonlinearly processed speech in reverberant spaces. This method is based on STI (speech transmission index), which is a popular measure of speech transmission quality of linearly processed signals. The proposed method, called broadband STI, is based on a broadband comparison of cumulated energy of the transmitted envelope modulation and the received modulation of the excitation signal. It can be applied to nonlinearly processed signals, like speech artificially slowed down for presenting in reverberant conditions.

K. Szklanny and J. Lachowicz in [7] describe a speech synthesis system, designed for a patient expecting laryngectomy for cancer. The system is based on the speech samples of the patient, recorded at the patient’s home before the surgery. The obtained speech synthesis model received good scores in listening tests performed by experts, even better than the model based on speech samples recorded for a professional voiceover actor.

### 2.2. Contributions in the Field of Image Techniques

Five papers in this Special Issue deal with image processing, including face images, microscopic images, and infrared images.

A. E. Mahdaoui, A. Ouahabi, and M. S. Moulay in [8] propose an image denoising method, called denoising-compressed sensing by regularizations terms. This method is intended to be applied in remote sensing and medical imaging, where the acquired image is compressed, and then reconstructed at the receiver side. The proposed method combines total variation regularization, which tends to excessively smooth images, and a non-local self-similarity constraint, which can restore high quality images. The tests on images with white Gaussian noise, and salt and pepper noise, confirmed improvement compared to other methods in terms of peak signal-to-noise ratio and structural similarity.

M. Geremek and K. Szklanny in [9] investigated deep learning based detection of genetic diseases from face images, for 15 genetic disorders associated with facial dysmorphism. The authors achieved 84% accuracy for 15 classes, and up to 96% for binary classification of particular diseases. Since there exist thousands of genetic diseases, similar systems can be implemented to automatically detect them, if sufficient training data are available.

In [10], E. Kubera, A. Kubik-Komar, K. Piotrowska-Weryszko, and M. Skrzypiec applied deep learning in an image recognition task, for the automatic classification of pollen grains into 3 classes. Pollen monitoring is most commonly based on counting pollen grains (for each species) found on adhesive tape in pollen traps. Such a counting, performed under a microscope, is a time-consuming task. In the reported work, deep convolutional neural network achieved 97.88% accuracy in the pollen grain recognition of birch, alder, and hazel. The proposed approach can support experts in their tedious work.

The next paper [11], written by E. Kubera, A. Kubik-Komar, P. Kurasiński, K. Piotrowska-Weryszko, and M. Skrzypiec, describes the application of deep neural network YOLO. (You Only Look Once) models for detection and recognition of pollen grains. The same species were used as in [10], i.e., birch, alder, and hazel. Since YOLO networks perform both recognition and detection, no image segmentation is needed as preprocessing, and multi-label images can be used as input data.

L. C. M. Dafico, E. Barreira, R. M. S. F. Almeida, and H. Carasek in [12] use infrared images for non-destructive moisture analysis of masonry walls. The authors compare infrared thermography with traditional techniques, and show both qualitative and quantitative analysis of the results obtained for two climate test chambers. A hot chamber, with an average temperature around 30 °C, and a cold chamber, with an average temperature around 15 °C were used. The correlation between infrared thermography and moisture content used are discussed in the paper, including the limitation of this technique, and future work needed to directly correlate temperature gradient to moisture content.

### 2.3. Contributions in the Field of Audiovisual Technologies

Five papers in this Special Issue present techniques related to the processing and usage of video signal and audiovisual data.

G. Canet Tarrés and M. Pardàs in [13] propose a technique for subtracting the background from video data, without annotation of any frames, and outputting the foreground mask. The presented methodology does not require dedicated training for each scene. The proposed technique uses a convolutional neural network as a refinement step of conventional background subtraction, and then an adversarial network to capture more details in the foreground mask. This approach can be applied in video surveillance systems.

In paper [14], F. Voss, S. Lyra, D. Blase, S. Leonhardt, and M. Lüken propose a low cost camera-based system for the monitoring of premature infants. The system uses a camera recording visible light, as well as two infrared thermography cameras. The tests were performed on a neonatal phantom, simulating pathologies such as hypothermia/hyperthermia and bradycardia/tachycardia. The implemented algorithm successfully measured heart rate and skin temperature, and the authors are planning further works, including movement simulation, as the fantom was lying still in the reported work.

K. Hirayama, S. Chen, S. Saiki, and M. Nakamura in [15] describe a system designed for detecting significant changes in facial expression that can be used in elderly care, based on the video signal from the built-in camera of the laptop. Facial feature data were extracted at 1 s intervals, thus creating a time series, for one of the authors and for five subjects receiving care. The proposed method successfully captured the moment of large facial movements, even for persons with poor facial expressions. The authors are planning to collect more facial data from elderly people in the future.

The study presented in [16] by J. Quan, Y. Miyake, and T. Nozawa, investigates automatic emotion recognition using visual, audio, and audio-visual features. The authors built two types of emotion recognition models: an individual model, and interpersonal model, capturing interpersonal interaction activities, both verbal and non-verbal. The individual model is based on such features as facial expression, gesture, and tone of voice, whereas interpersonal model is based on synchronization with interlocutor, e.g., mutual gaze, body and speech synchronization. The authors found that the interpersonal model outperformed the individual models.

K. Szklanny, M. Wichrowski, and A. Wieczorkowska in [17] present two applications for persons with aphasia, to facilitate communication and sharing their daily experiences by using visual storytelling forms composed of photos, videos, etc. The authors performed usability tests, supervised by a neuropsychologist, on six participants with aphasia who were able to communicate. The tests indicate that the persons with aphasia would like to use these applications to improve the quality of their lives through storytelling methods, and the appropriate selection of screen layout, limited functionalities, and simple workflow of interactions fulfil the needs of these users.

## 3. Conclusions

The papers collected in this Special Issue present a wide range of topics in audio, image, and video domains. I do hope the readership of *Sensors* will find many of them interesting and inspiring.

I would like to thank all the authors who submitted papers to this Special Issue, the reviewers for their efforts in improving the papers, and the editors of *Sensors*, for their hard work in successful preparation of this Special Issue.
